# Subpopulations of somatostatin-immunoreactive non-pyramidal neurons in the amygdala and adjacent external capsule project to the basal forebrain: evidence for the existence of GABAergic projection neurons in the cortical nuclei and basolateral nuclear complex

**DOI:** 10.3389/fncir.2012.00046

**Published:** 2012-07-24

**Authors:** Alexander J. McDonald, Franco Mascagni, Violeta Zaric

**Affiliations:** Department of Pharmacology, Physiology, and Neuroscience, University of South Carolina School of MedicineColumbia, SC, USA

**Keywords:** amygdala, basal forebrain, GABAergic projection neurons, somatostatin, neuropeptide Y, calbindin, Fluorogold

## Abstract

The hippocampus and amygdala are key structures of the limbic system whose connections include reciprocal interactions with the basal forebrain (BF). The hippocampus receives both cholinergic and GABAergic afferents from the medial septal area of the BF. Hippocampal projections back to the medial septal area arise from non-pyramidal GABAergic neurons that express somatostatin (SOM), calbindin (CB), and neuropeptide Y (NPY). Recent experiments in our lab have demonstrated that the basolateral amygdala, like the hippocampus, receives both cholinergic and GABAergic afferents from the BF. These projections arise from neurons in the substantia innominata (SI) and ventral pallidum (VP). It remained to be determined, however, whether the amygdala has projections back to the BF that arise from GABAergic non-pyramidal neurons. This question was investigated in the present study by combining Fluorogold (FG) retrograde tract tracing with immunohistochemistry for GABAergic non-pyramidal cell markers, including SOM, CB, NPY, parvalbumin, calretinin, and glutamic acid decarboxylase (GAD). FG injections into the BF produced a diffuse array of retrogradely labeled neurons in many nuclei of the amygdala. The great majority of amygdalar FG+ neurons did not express non-pyramidal cell markers. However, a subpopulation of non-pyramidal SOM+ neurons, termed “long-range non-pyramidal neurons” (LRNP neurons), in the external capsule, basolateral amygdala, and cortical and medial amygdalar nuclei were FG+. About one-third of the SOM+ LRNP neurons were CB+ or NPY+, and one-half were GAD+. It remains to be determined if these inhibitory amygdalar projections to the BF, like those from the hippocampus, are important for regulating synchronous oscillations in the amygdalar-BF network.

## Introduction

The amygdala is one of the most important brain regions for the generation of emotional behavior and in the formation and retrieval of emotional memories, particularly those related to fear and anxiety (Sah et al., 2003; Pape and Pare, 2010). Stimulation of the amygdala produces a wide range of behaviors, including aggressive behavior and flight, feeding and drinking, and sexual activity (Kaada, 1972). However, it is often overlooked that the most common response to amygdalar stimulation is an orientation/arousal response that is indistinguishable from that produced by stimulation of the reticular formation and is associated with cortical desynchronization (Kaada, 1972). This orientation/arousal response is seen as the initial phase of all behavioral responses elicited by amygdalar stimulation and is dependent on the projections of the amygdala to the basal forebrain (BF) (Kaada, 1972; Dringenberg and Vanderwolf, 1996).

The BF contains a diffuse array of cholinergic and noncholinergic neurons that extends through a continuous region which includes the medial septal area, diagonal band of Broca, ventral pallidum (VP), and substantia innominata (SI) (Mesulam et al., 1983; Woolf, 1991). This complex has topographically organized connections with different forebrain regions including the hippocampus, neocortex, and amygdala (Mesulam et al., 1983; Zaborszky et al., 1999). Recent studies in our laboratory (Mascagni and McDonald, 2009; McDonald et al., 2011; Muller et al., 2011) indicate that the projections of the amygdala to the SI and ventral pallidal portions of the BF (SI/VP region) share certain organizational features with hippocampal and prefrontal cortical projections to the portions of the BF associated with these areas (medial septum and SI, respectively). Thus, in all three areas the cholinergic BF neurons innervate both pyramidal cells and GABAergic non-pyramidal cells (Frotscher and Léránth, 1985; Beaulieu and Somogyi, 1991; Henny and Jones, 2008; Muller et al., 2011), whereas the axons of GABAergic BF neurons form multiple synaptic contacts with individual GABAergic non-pyramidal cells (Freund and Gulyás, 1991; Freund and Meskenaite, 1992; Freund and Buzsáki, 1996; McDonald et al., 2011).

An interesting aspect of the hippocampal projections back to the medial septal region of the BF is that these projections arise mostly from GABAergic non-pyramidal cells that mainly target the GABAergic BF neurons that project to the hippocampus (Alonso and Köhler, 1982; Tóth and Freund, 1992; Tóth et al., 1993; Zappone and Sloviter, 2001; Jinno and Kosaka, 2002; Gulyás et al., 2003; Jinno et al., 2007). These GABAergic hippocamposeptal neurons are thought to be critical for the generation of rhythmic oscillations in the hippocampus (Tóth et al., 1993; Dragoi et al., 1999; Wang, 2002; Jinno et al., 2007). Since oscillatory activity in the amygdala is important for emotional arousal and emotional memory (Paré and Collins, 2000; Paré et al., 2002; Pape et al., 2005; Lesting et al., 2011), it is important to determine if there are also GABAergic non-pyramidal neurons in the amygdala that project to the BF. This question was investigated in the present study by combining Fluorogold (FG) retrograde tract tracing with immunohistochemistry for GABAergic non-pyramidal cell markers, including somatostatin (SOM), calbindin (CB), neuropeptide Y (NPY), parvalbumin (PV), calretinin (CR), and glutamic acid decarboxylase (GAD, the synthetic enzyme for GABA).

## Materials and methods

### Injections and tissue preparation

A total of 15 adult male Sprague-Dawley rats (250–350 g; Harlan, Indianapolis, IN) received injections of FG into the portions of the BF that give rise to the cholinergic innervation of the amygdala, including the SI and VP, or into portions of the striatum that are dorsally adjacent to the BF. All experiments were carried out in accordance with the National Institutes of Health Guide for the Care and Use of Laboratory Animals and were approved by the Institutional Animal Use and Care Committee (IACUC) of the University of South Carolina. All experiments were conducted in a manner that minimized suffering and the number of animals used.

Rats were anesthetized with sodium pentobarbital (50 mg/kg) and placed in a stereotaxic head holder (Stoelting, Wood Dale, IL) for injections of 2% FG (hydroxystilbamidine; Invitrogen, Carlsbad, CA) into the SI/VP region or adjacent striatum using coordinates obtained from an atlas of the rat brain (Paxinos and Watson, 1997). Unilateral (*n* = 13 rats) or bilateral (*n* = 2 rats) iontophoretic injections of FG in saline were made via glass micropipettes (40 μ m inner tip diameter) using a Midgard high voltage current source set at 1.0–2.0 μ A (7 s on, 7 s off, for 20–40 min). Micropipettes were left in place for 10 min, and then slowly withdrawn with the current reversed to prevent FG from flowing up the pipette track. After a 5-day survival, 13 of the 15 rats were anesthetized with chloral hydrate (350 mg/kg) and perfused intracardially with phosphate buffered saline (PBS; pH 7.4) containing 1.0 % sodium nitrite (50 ml), followed by 4.0% paraformaldehyde in 0.1 M phosphate buffer at pH 7.4 (500 ml). After a 5-day survival, two of the 15 rats with unilateral FG injections were anesthetized and received intracerebroventricular injections of colchicine (50 μ g dissolved in 5.0 μ l saline into each lateral ventricle); one day later, these two rats were anesthetized and perfused with 4.0% paraformaldehyde. Following perfusion, all brains were removed and postfixed for 3.5 h in 4.0% paraformaldehyde. Brains were sectioned on a vibratome at a thickness of 50 μ m in the coronal plane and processed for immunohistochemistry. All antibodies were diluted in a solution containing 1% normal goat serum, 0.4% Triton-X 100, and 0.1 M PBS.

### Triple-labeling experiments

In six rats with unilateral injections of the BF, two series of sections through the amygdala at 200–300 μ m intervals were incubated in one of two different primary antibody cocktails overnight at 4°C: (1) an anti-FG/SOM/NPY cocktail, or (2) an anti-FG/SOM/CB cocktail. The following primary antibodies were used: (1) a polyclonal FG antibody raised in guinea pig (1:3000; donated by Dr. Lothar Jennes, University of Kentucky); (2) a monoclonal SOM antibody raised in mouse (1:4000; donated by Dr. Alison Buchan, University of British Columbia); (3) a polyclonal NPY antibody raised in rabbit (1:3000; Bachem Americas, Torrance, CA); and (4) a polyclonal CB antibody raised in rabbit (1:6000; donated by Dr. Kenneth Baimbridge, University of British Columbia). After incubation in the primary antibody cocktails, sections were rinsed in three changes of PBS (10 min each) and then incubated in a cocktail of three secondary antibodies for 3 h at room temperature (1:400; Invitrogen, Eugene, OR): (1) goat anti-guinea pig Alexa-488; (2) goat anti-mouse Alexa-546; and (3) goat anti-rabbit Alexa-633. Sections were then rinsed in three changes of PBS (10 min each) and mounted on glass slides using Vectashield mounting medium (Vector Laboratories, Burlingame, CA).

The two colchicine-injected rats were used for colocalization of FG, SOM, and GAD67. It is well-established that the somata of GABAergic projection neurons, including GABAergic hippocamposeptal projection neurons, often contain levels of GAD and GABA that are below the threshold for immunohistochemical detection (Tóth and Freund, 1992). This is thought to be due to slow turnover of GAD/GABA combined with rapid transport of GAD/GABA to axon terminals via axonal transport. It was hoped that colchicine injections would increase somatic levels of GAD by disrupting microtubules involved in axonal transport. In each rat, a series of sections through the amygdala at 100 μ m intervals was incubated in a primary antibody cocktail at 4°C containing a polyclonal FG antibody raised in guinea pig (1:3000; donated by Dr. Lothar Jennes, University of Kentucky), a polyclonal SOM antibody raised in rabbit (1:6000; Bachem Americas, Torrance, CA), and a monoclonal GAD67 antibody raised in mouse (1:300; Millipore, Billerica, MA). To further enhance immunostaining for GAD, incubation in the primary antibody cocktail was extended to 4 days. Sections were then rinsed in three changes of PBS (10 min each) and incubated in a cocktail of three secondary antibodies for 3 h at room temperature (Invitrogen, Eugene, OR): (1) goat anti-guinea pig Alexa-488 (1:400); (2) goat anti-mouse Alexa-546 (1:200); and (3) goat anti-rabbit Alexa-633 (1:400). Sections were then rinsed in three changes of PBS (10 min each) and mounted on glass slides using Vectashield.

In all brains, a separate series of sections through the injection sites and amygdala at 150–300 μ m intervals was incubated in either the guinea pig FG antibody (1:5000) or a rabbit FG antibody (1:8000; Millipore) overnight at 4°C. These sections were then processed for avidin–biotin peroxidase immunohistochemistry using a guinea pig or rabbit Vectastain ABC kit (Vector Laboratories). Nickel-enhanced DAB (3, 3′-diaminobenzidine-4HCl, Sigma Chemical Co., St. Louis, MO) was used as a chromogen to generate a black reaction product (Hancock, 1986). These sections were mounted on gelatinized slides, dried overnight, counterstained with pyronin Y (a pink Nissl stain), dehydrated in ethanol, cleared in xylene, and coverslipped with Permount (Fisher Scientific, Pittsburgh, PA).

### Double-labeling experiments

Double-labeling experiments were performed to determine if PV+ or CR+ amygdalar neurons in the amygdala project to the BF (*n* = 4 rats), and if SOM+, PV+, or CR+ amygdalar neurons had projections to portions of the striatum that were adjacent to the BF (*n* = 3 rats). The latter cases were controls for some of the BF injections that also involved the adjacent striatum. In four rats with unilateral (*n* = 2 rats) or bilateral injections (*n* = 2 rats) of the BF, and three rats with unilateral injections of the striatum, three series of sections through the amygdala at 150–300 μ m intervals were incubated in one of three different primary antibody cocktails overnight at 4°C: (1) an anti-FG/SOM cocktail, (2) an anti-FG/PV cocktail, or (2) an anti-FG/CR cocktail. The following primary antibodies were used: (1) a polyclonal FG antibody raised in guinea pig (1:3000; donated by Dr. Lothar Jennes, University of Kentucky); (2) a monoclonal SOM antibody raised in mouse (1:4000; donated by Dr. Alison Buchan, University of British Columbia); (3) a monoclonal PV antibody raised in mouse (1:5000; Sigma); and (4) a monoclonal CR antibody raised in mouse (1:2000; Millipore). After incubation in the primary antibody cocktails, sections were rinsed in three changes of PBS (10 min each) and then incubated in a cocktail of goat anti-guinea pig Alexa-488 and goat anti-mouse Alexa-546 antibodies for 3 h at room temperature (1:400; Invitrogen). Sections were then rinsed in three changes of PBS (10 min each) and mounted on glass slides using Vectashield mounting medium (Vector Laboratories). In all brains, a separate series of sections through the BF and amygdala at 150–300 μ m intervals was incubated in either a guinea pig FG antibody (1:5000) or a rabbit FG antibody (1:8000; Millipore, Billerica, MA) overnight at 4°C and processed for avidin–biotin peroxidase immunohistochemistry as described above.

### Analysis

Injection sites (processed for immunoperoxidase) were mapped onto template drawings taken from an atlas of the rat brain (Paxinos and Watson, 1997) using a drawing tube attached to an Olympus BX51 microscope under bright-field illumination. Amygdalar sections processed for immunofluorescence were examined with a Zeiss LSM 510 Meta confocal microscope. Fluorescence of Alexa-488, Alexa-546, and Alexa-633 dyes was analyzed using filter configurations for sequential excitation/imaging via 488, 543, and 633 nm channels. In the 13 noncolchicine-injected brains, FG-labeled neurons exhibiting neuronal marker immunostaining were plotted at 0.5 mm intervals through the amygdala (bregma levels −1.8, −2.3, −2.8, −3.3, −3.8) onto template drawings taken from an atlas of the rat brain (Paxinos and Watson, 1997). The location of labeled neurons at each level was determined using anatomical cues, including the staining patterns of the neuronal markers, which differed in the various nuclei of the amygdala. Control sections were processed with one of the antibodies omitted from the primary antibody cocktail; in all cases, only the labeling with the secondary fluorescent antibodies corresponding to the nonomitted primary antibodies was observed, and only on the appropriate channel. These results indicated that the secondary antibodies were specific for guinea pig, rabbit, or mouse immunoglobulins, and that there was no “crosstalk” between channels (Wouterlood et al., 1998). Digital images were adjusted for brightness and contrast using Photoshop 6.0 software.

The distribution of amygdalar FG+/SOM+ neurons in the two colchicine-injected brains (reacted for FG, SOM, and GAD) appeared to be identical to that seen in the noncolchicine-injected brains, but the exact positions of these neurons were not plotted. Instead, each amygdalar nucleus was examined to determine if any of the FG+/SOM+ neurons was GAD+. In addition, the posterior basomedial nucleus (BMp) in both brains was subjected to a more extensive quantitative analysis. In 11 sections in brain R46, and 10 sections in brain R47, counts of FG+ single-labeled neurons, FG+/SOM+ double-labeled neurons, and FG+/SOM+/GAD+ triple-labeled neurons were counted in a field (400 × 400 μ m) in the center of the nucleus at 200X magnification.

## Results

### FG injection sites into the basal forebrain and the pattern of retrogradely labeled neurons in the amygdala

**Figure 1:**
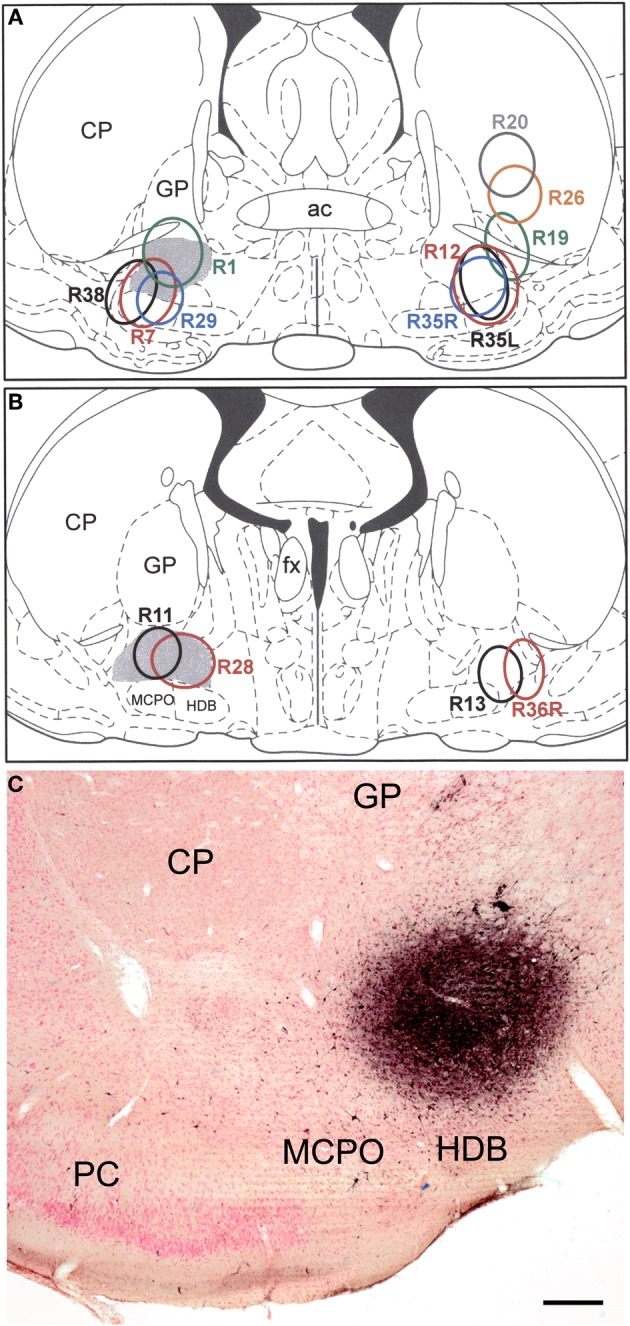
**(A** and **B)** Drawings showing the FG injection sites into the BF (and overlying striatum) plotted at the Bregma −0.3 **(A)** and Bregma −0.8 **(B)** levels of the atlas by Paxinos and Watson (1997). Gray shading indicates the SI/VP portion of the BF, the main source of the cholinergic innervation of the amygdala. Injections in rats whose brains were processed for triple-labeling immunofluorescence (FG/SOM/NPY and FG/SOM/CB) are depicted on the left. Injections in rats whose brains were processed for double-labeling immunofluorescence (FG/SOM, FG/PV, and FG/CR) are depicted on the right. The letters “L” (left) and “R” (right) in three of the case numbers (R35L, R35R, R36R) indicate whether the injection site in these three bilaterally injected rats was on the left or right side. **(C)** Photomicrograph of the injection site in case R28. This section was counterstained with pyronin Y (a pink Nissl stain). Left is lateral. Abbreviations: ac, anterior commissure; CP, caudate-putamen; fx, fornix; GP, globus pallidus; HDB, horizontal limb of the nucleus of the diagonal band; MCPO, magnocellular preoptic nucleus; PC, piriform cortex. Scale bar in *C* = 250 μ m.

The injection sites into the SI/VP portion of the BF, the main source of the cholinergic innervation of the amygdala (Carlsen et al., 1985), collectively involved virtually all portions of the SI/VP complex (Figure 1). Several of the injections also spread into the ventrally adjacent magnocellular preoptic nucleus or impinged slightly on the laterally adjacent ventral caudate-putamen (i.e., fundus striati).

**Figure 2:**
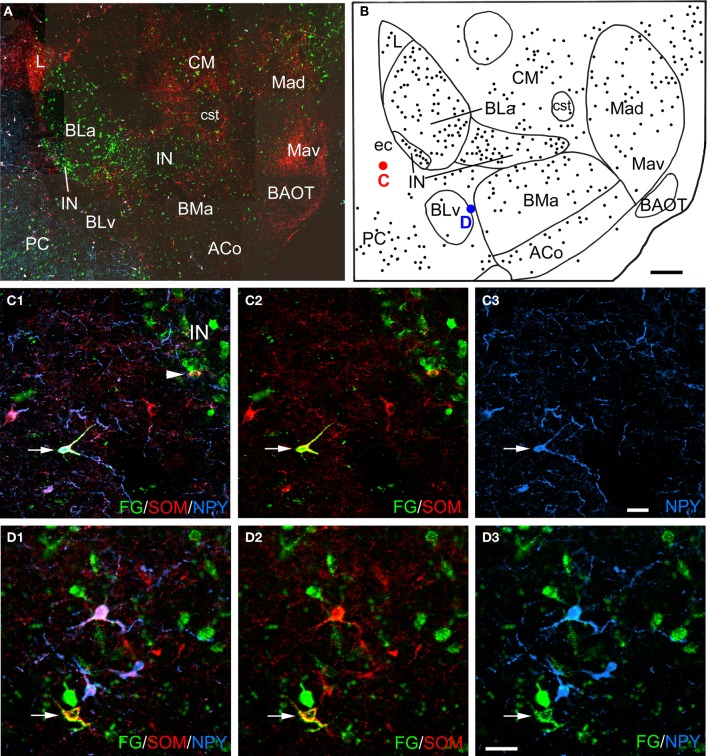
**(A)** Low power photomontage of a coronal section through the rostral amygdala (Bregma −2.1) in case R7 that was triple-labeled for FG (green), SOM (red), and NPY (blue). Lateral is left. After this section was analyzed the coverslip was removed and the section was counterstained with pyronin Y. This allowed the nuclear borders to be more precisely defined, as depicted in **B**. **(B)** Drawing of the distribution of FG-labeled neurons seen in **A**, created by overlaying a plot of FG-labeled neurons in this section over a drawing of the nuclei identified after counterstaining. The positions of a triple-labeled FG/SOM/NPY+ neuron in the external capsule (ec, red dot), and a FG/SOM+ neuron located between the ventral basolateral nucleus (BLv) and the anterior basomedial nucleus (BMa) that was NPY-negative (blue dot) are indicated. **(C1–C3)** Photomicrographs of the FG/SOM/NPY+ neuron shown in red in **B** (arrow). Also note a FG/SOM+ neuron (yellow) that was NPY-negative at the lateral edge of the lateral intercalated nucleus (IN; arrowhead in **C1**). **(C1)** Merged image visualizing all three channels. **(C2)** Image with the blue channel omitted, showing that the neuron exhibited colocalization of FG and SOM (yellow). **(C3)** Image with only the blue channel, showing that the neuron was also NPY+ (blue). **(D1–D3)** Photomicrographs of the FG/SOM+ neuron shown in blue in **B** (arrow). **(D1)** Merged image visualizing all three channels. **(D2)** Image with the blue channel deleted, showing that the neuron exhibited colocalization of FG and SOM (yellow). **(D3)** Image with the red channel deleted, showing that the neuron was NPY-negative. Also note that the three blue NPY neurons located above the FG/SOM+ neuron colocalize SOM (see **D1** and **D2**). Additional abbreviations: ACo, anterior cortical nucleus; BAOT, bed nucleus of the accessory olfactory tract; BLa, anterior basolateral nucleus; CM, medial central nucleus; cst, commissural stria terminalis; L, lateral nucleus; Mad, anterodorsal medial nucleus; Mav, anteroventral medial nucleus; PC, piriform cortex. Scale bars = 300 μ m in **B** (also applies to **A**), 40 μ m in **C** and **D**.

**Figure 3:**
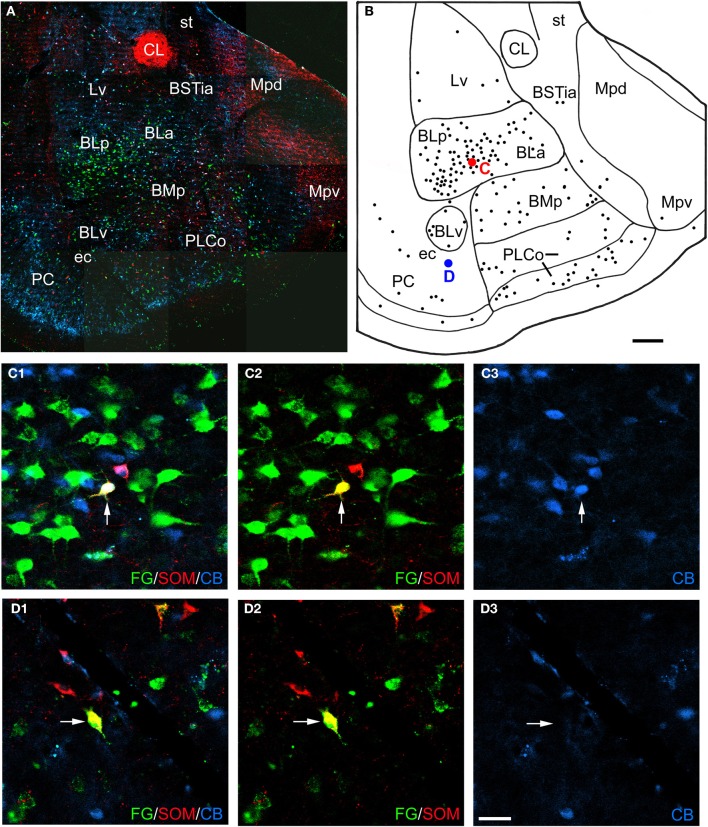
**(A)** Low power photomontage of a coronal section through the middle of the amygdala (Bregma −3.3) in case R7 that was triple-labeled for FG (green), SOM (red), and CB (blue). Lateral is left. After this section was analyzed the coverslip was removed and the section was counterstained with pyronin Y. This allowed the nuclear borders to be more precisely defined, as depicted in **B**. **(B)** Drawing of the distribution of FG-labeled neurons seen in **A**, created by overlaying a plot of FG-labeled neurons in this section over a drawing of the nuclei identified after counterstaining. The positions of a triple-labeled FG/SOM/CB+ neuron in the basolateral nucleus (red dot), and a FG/SOM+ neuron in the external capsule (ec) that was CB-negative (blue dot) are indicated. **(C1–C3)** Photomicrographs of the FG/SOM/CB+ neuron shown in red in **B** (arrow). **(C1)** Merged image visualizing all three channels. **(C2)** Image with the blue channel omitted, showing that the neuron exhibited colocalization of FG and SOM (yellow). **(C3)** Image with only the blue channel, showing that the neuron was also CB+ (blue). **(D1–D3)** Photomicrographs of the FG/SOM+ neuron shown in blue in **B** (arrow). **(D1)** Merged image visualizing all three channels, showing that the neuron exhibited colocalization of FG and SOM (yellow). **(D2)** Image with the blue channel deleted, showing that the neuron exhibited colocalization of FG and SOM (yellow). **(D3)** Image with the green and red channels deleted, showing that the neuron was CB-negative. Additional abbreviations: BLa, anterior basolateral nucleus; BLp, posterior basolateral nucleus; BMp, posterior basomedial nucleus; BSTia, intra-amygdalar portion of the bed nucleus of the stria terminalis; CL, lateral central nucleus; Lv, ventromedial lateral nucleus; Mpd, posterodorsal medial nucleus; Mpv, posteroventral medial nucleus; PLCo, posterolateral cortical nucleus; PC, piriform cortex; st, stria terminalis. Scale bars = 300 μ m in **B** (also applies to **A**), 40 μ m in **D** (also applies to **C**).

Examination of sections stained for immunofluorescence or immunoperoxidase revealed that the distribution of retrogradely labeled FG+ neurons in the amygdala was similar in most of the cases examined in this study and consisted of a diffuse array of neurons extending through many nuclei of the ipsilateral amygdala. At rostral levels of the amygdala scattered FG+ neurons were seen in most amygdalar nuclei, including the basolateral, anterior basomedial, anterior cortical, central, medial, and intercalated nuclei, as well as in the laterally adjacent ventral portion of the external capsule (Figures 2A,B). At middle levels of the amygdala scattered FG+ neurons were seen in the basolateral, posterior basomedial, and posterolateral cortical nuclei, as well as in the ventral portion of the external capsule (Figures 3A,B). Little retrograde labeling was seen, however, in the lateral or medial nuclei at this level. At caudal levels of the amygdala scattered FG+ neurons were seen in the posterior basolateral, posterior basomedial, and posterolateral cortical nuclei, as well as in the amygdalohippocampal area and the adjacent external capsule. Few neurons were seen in the lateral nucleus at caudal levels of the amygdala.

Injection sites that impinged on the fundus striati (e.g., R38, R12, R35L, and R35R) had a significantly higher density of neurons in the basolateral nucleus than cases that did not involve this striatal region. In contrast, in case R28, the most caudomedial of the injections, there were almost no FG+ neurons in the basolateral or lateral nuclei, but there was a higher density of FG+ neurons in the lateral subdivision of the central nucleus than was seen in other cases. In cases with unilateral injections that involved the SI/VP complex, without spread into the adjacent striatum, there were usually only about 0–8 lightly labeled FG+ neurons per section in the contralateral amygdala that were distributed in the same nuclei that were labeled ipsilaterally. Injections into the BF that also involved the adjacent striatum exhibited significant retrograde labeling in the contralateral basolateral nucleus (anterior, posterior, and ventral subdivisions) and nucleus of the lateral olfactory tract.

### Colocalization of interneuronal markers with FG

Amygdalar sections from six rats with unilateral injections of FG into the BF were processed for both FG/SOM/NPY and FG/SOM/CB triple-labeling immunofluorescence histochemistry (see left sides of Figures 1A and B for the location of these injection sites). In all six rats, there were a small number of nonpyramidal FG+ neurons in the amygdala and adjacent external capsule that exhibited immunoreactivity for one or more nonpyramidal cell markers; these neurons will be termed “long-range non-pyramidal neurons” (LRNP neurons) in the present account. Figure 2 shows examples from case R7 of a FG/SOM/NPY+ triple-labeled neuron in the external capsule (Figure 2C) and a FG/SOM+ neuron that did not exhibit NPY-ir located at the border separating the ventral basolateral nucleus from the anterior basomedial nucleus (Figure 2D). Figure 3 shows examples from case R7 of a FG/SOM/CB+ triple-labeled neuron in the basolateral nucleus (Figure 3C) and a FG/SOM+ neuron that did not exhibit CB-ir located in the ventral part of the external capsule (Figure 3D). In all cases, these LRNP neurons exhibited a non-pyramidal morphology. They had medium-sized cell bodies (15–20 μ m in diameter) and 3–4 primary dendrites.

**Figure 4:**
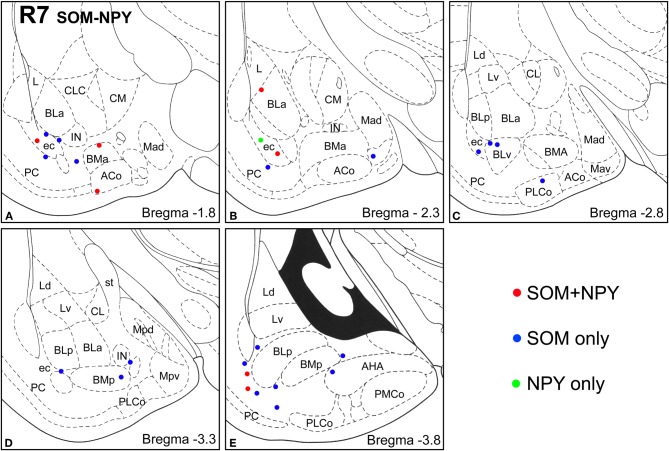
**Sections arranged from rostral (A) to caudal (E) depicting the locations of long-range non-pyramidal neurons in the amygdalar region expressing SOM and/or NPY in case R7.** Each dot represents one neuron. Red dots are FG+ neurons expressing SOM and NPY. Blue dots are FG+ neurons expressing SOM, but not NPY. Green dots are FG+ neurons expressing NPY, but not SOM. Templates are modified from the atlas by Paxinos and Watson (1997).

**Figure 5:**
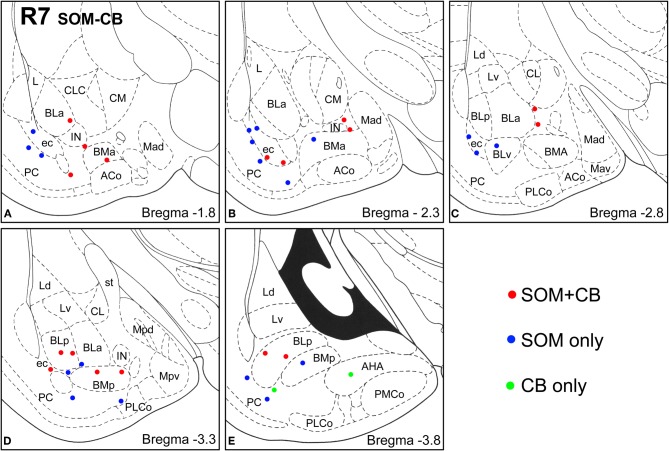
**Sections arranged from rostral (A) to caudal (E) depicting the locations of long-range non-pyramidal neurons in the amygdalar region expressing SOM and/or CB in case R7.** Each dot represents one neuron. Red dots are FG+ neurons expressing SOM and CB. Blue dots are FG+ neurons expressing SOM, but not CB. Green dots are FG+ neurons expressing CB, but not SOM. Templates are modified from the atlas by Paxinos and Watson (1997).

**Figure 6:**
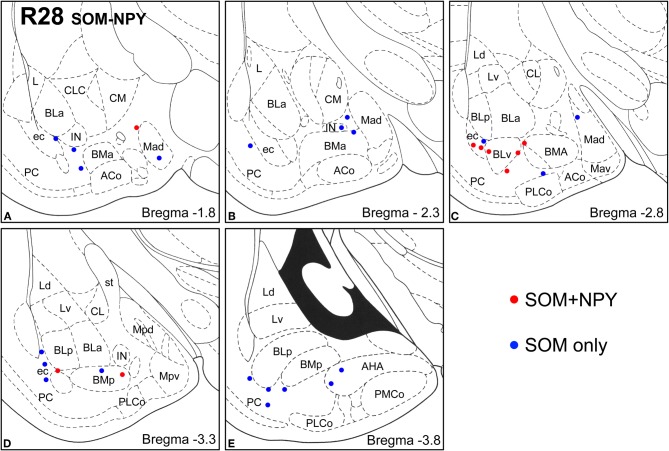
**Sections arranged from rostral (A) to caudal (E) depicting the locations of long-range non-pyramidal neurons in the amygdalar region expressing SOM and NPY in case R28.** Each dot represents one neuron. Red dots are FG+ neurons expressing SOM and NPY. Blue dots are FG+ neurons expressing SOM, but not NPY. Templates are modified from the atlas by Paxinos and Watson (1997).

**Figure 7:**
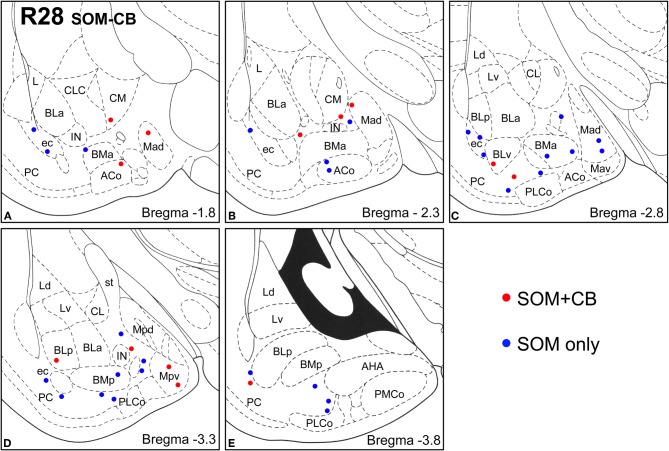
**Sections arranged from rostral (A) to caudal (E) depicting the locations of long-range non-pyramidal neurons in the amygdalar region expressing SOM and CB in case R28.** Each dot represents one neuron. Red dots are FG+ neurons expressing SOM and CB. Blue dots are FG+ neurons expressing SOM, but not CB. Templates are modified from the atlas by Paxinos and Watson (1997).

LRNP neurons were always found interspersed among single-labeled FG+ neurons in the various nuclei of the amygdala, but they were always far outnumbered by FG+ neurons that did not express the non-pyramidal cell markers studied. For example, the section through the rostral amygdala from case R7 that is depicted in Figures 2A and B contained 7 LRNP neurons (either FG/SOM+ or FG/SOM/NPY+), and 138 single-labeled FG+ neurons in the area containing the lateral nucleus, basolateral nucleus, basomedial nucleus, cortical nucleus, and external capsule. Thus, 4.8% (7/145) of the projection neurons in this region were LRNP neurons in this section. The section through the middle amygdala from case R7 depicted in Figures 3A and B contained 9 LRNP neurons (either FG/SOM+ or FG/SOM/CB+) and 152 single-labeled FG+ neurons in the area containing the basolateral nucleus, basomedial nucleus, cortical nucleus, and external capsule. Thus, 5.6% (9/161) of the projection neurons in this region were LRNP neurons in this section. Most amygdalar sections in the cases examined in this study contained 4–10 LRNP neurons (Figures 4–7, 9). Although no formal cell counts of single-labeled FG+ neurons were made in all of these sections, it appeared that their number approximated that seen in R7 (Figures 2B, 3B). Thus, LRNP neurons appear to constitute about 5% of the amygdalar projection neurons labeled by injections of FG into the BF.

Virtually all LRNP neurons expressed SOM; very few contained only NPY or CB (Figures 4–7, Tables 1 and 2). However, these SOM+ neurons labeled with FG constituted a small subpopulation of the total SOM+ neuronal population in each amygdalar nucleus. Cell counts pooled from three cases revealed that 35% (29/83) of SOM+ LRNP neurons also expressed NPY, whereas 37% (33/89) also expressed CB (Tables 1 and 2). There was a concentration of LRNP neurons in the ventral part of the external capsule (ec) located just lateral to the ventral portions of the basolateral nuclear complex (Figures 4–7). Most of the other LRNP neurons were found in ventral portions of the amygdala, including the ventral basolateral nucleus, anterior and posterior basomedial nuclei, and the anterior and posterolateral cortical nuclei (Figures 4–7). There was no anatomical segregation of double-labeled FG/SOM+ neurons from triple-labeled FG/SOM/NPY+ (Figures 4 and 6) or FG/SOM/CB+ neurons (Figures 5 and 7); all were intermingled in ventral portions of the amygdala and external capsule.

**Table 1:** **Cell counts of double and triple-labeled neurons in the amygdala in sections labeled for FG, SOM, and NPY**.

**Case**	**FG+/SOM+ Double-labeled neurons**	**FG+/NPY+ Double-labeled neurons**	**FG+/SOM+/NPY+ Triple-labeled neurons**
R38	14	0	13
R7	19	1	7
R28	21	0	9
Total	54	1	29

**Table 2:** **Cell counts of double and triple-labeled neurons in the amygdala in sections labeled for FG, SOM, and CB**.

**Case**	**FG+/SOM+ Double-labeled neurons**	**FG+/CB+ Double-labeled neurons**	**FG+/SOM+/CB+ Triple-labeled neurons**
R38	15	0	5
R7	16	2	16
R28	26	0	12
Total	56	2	33

**Figure 8:**
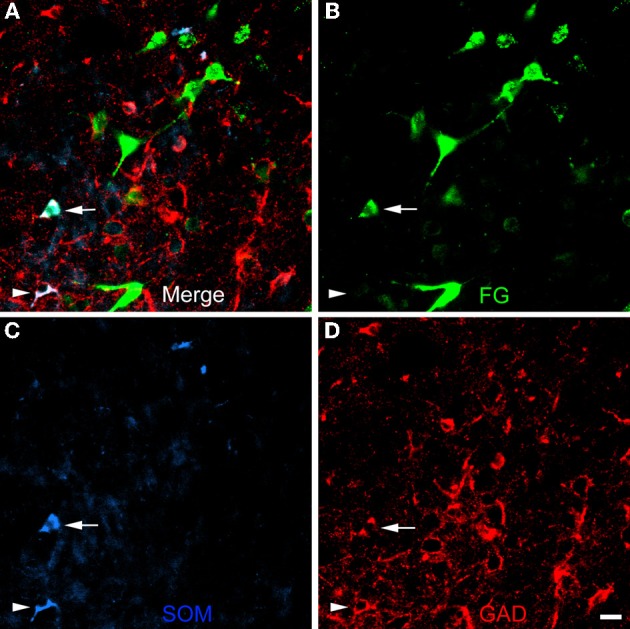
**Photomicrographs of neurons in the posterior subdivision of the basomedial nucleus (BMp) at bregma level-3.3 in a section triple-labeled for FG (green), SOM (blue), and GAD (red). (A)** Merged image visualizing all three channels. Arrow indicates a neuron triple-labeled for FG/SOM/GAD+. Arrowhead indicates a SOM/GAD+ neuron that is not retrogradely labeled with FG. The locations of the same neurons are indicated by identical arrows and arrowheads in **B**, **C**, and **D**. **(B)** Green channel image showing FG+ neurons. **(C)** Blue channel image showing SOM+ neurons. **(D)** Red channel image showing GAD+ neurons. Scale bar in *D* = 20 μ m (also applies to **A**, **B**, and **C**).

**Figure 9:**
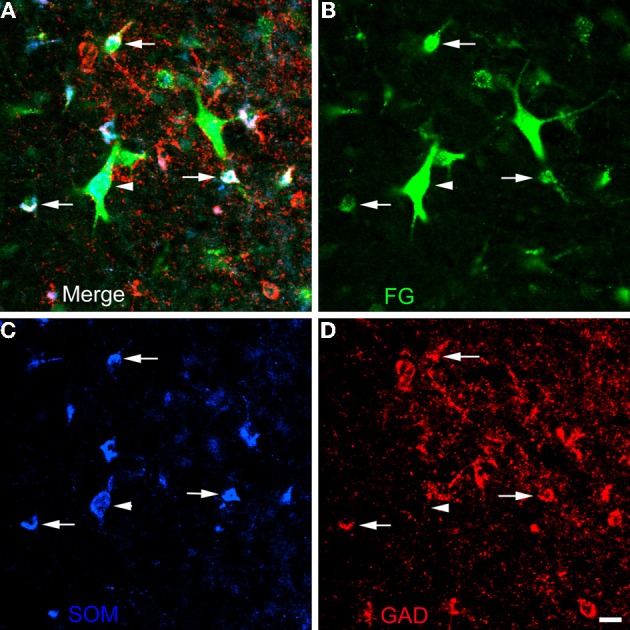
**Photomicrographs of neurons in the external capsule laterally adjacent to the ventral basolateral nucleus at bregma level-2.8 in a section triple-labeled for FG (green), SOM (blue), and GAD (red). (A)** Merged image visualizing all three channels. Arrows indicate 3 of 6 neurons in this field triple-labeled for FG/SOM/GAD+ (white). Arrowhead indicates a GAD-negative FG/SOM+ neuron (blue-green). The locations of the same neurons are indicated by identical arrows and arrowheads in **B**, **C**, and **D**. **(B)** Green channel image showing FG+ neurons. **(C)** Blue channel image showing SOM+ neurons. **(D)** Red channel image showing GAD+ neurons. Scale bar in *D*= 20 μ m (also applies to **A**, **B**, and **C**).

The unilateral FG injection sites in both of the colchicine-injected rats (not shown) closely approximated that seen in case R7 (see Figure 1), and the distribution of FG+ single-labeled neurons and LRNP neurons in these FG/SOM/GAD preparations was similar to that described above for the FG/SOM/CB and FG/SOM/NPY preparations. FG/SOM/GAD+ triple-labeled LRNP neurons were observed in the basomedial nuclei (Figure 8), cortical nuclei, ventral external capsule (Figure 9), and to a much lesser extent in the basolateral nucleus. No SOM-negative FG/GAD+ neurons were seen in these nuclei. Cell counts of labeled neurons in the posterior subdivision of the basomedial nucleus (BMp; Table 3) revealed that 13.0% (78/598) of FG+ neurons were LRNP neurons (either FG/SOM+ neurons or FG/SOM/GAD+ neurons). Approximately one-half of these LRNP neurons (53.8%, 42/78) were GAD+ (Table 3). The cortical nucleus appeared to have similar ratios of labeled neurons, but no quantitation was performed. GAD staining in the neuropil of the central and medial nuclei was intense, but little if any staining was seen in cell bodies, with the exception of a few scattered neurons in the central nucleus. Because of the lack of suitable GAD staining in neuronal somata, all FG/SOM+ neurons observed in these nuclei were GAD-negative.

**Table 3:** **Cell counts of labeled neurons in the posterior subdivision of the basomedial amygdalar nucleus in sections labeled for FG, SOM, and GAD**.

**Case**	**FG+ Single-labeled neurons**	**FG+/SOM+ Double-labeled neurons**	**FG+/SOM+/GAD+ Triple-labeled neurons**
R46	298	17	25
R47	222	19	17
Total	520	36	42

**Figure 10:**
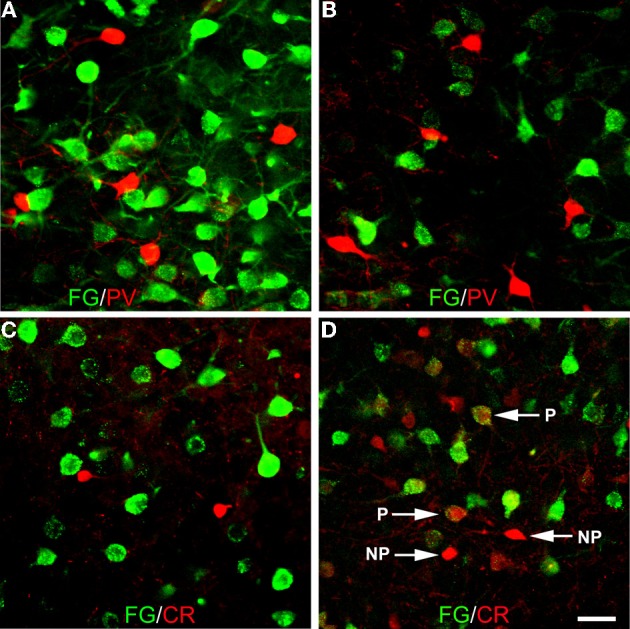
**Photomicrographs of immunofluorescence preparations dual-labeled for FG/PV or FG/CR. (A)** Dual-localization of FG (green) and PV (red) in the anterior basolateral nucleus. Note lack of colocalization. **(B)** Dual-localization of FG (green) and PV (red) in the posterior basomedial nucleus. Note lack of colocalization. **(C)** Dual-localization of FG (green) and CR (red) in the anterior basolateral nucleus. Note lack of colocalization. **(D)** Dual-localization of FG (green) and CR (red) in the posterior basomedial nucleus. Note lack of colocalization in small non-pyramidal neurons exhibiting robust CR-ir (NP), but existence of colocalization in larger pyramidal neurons exhibiting light CR-ir (P). Scale bar in *D*= 40 μ m (also applies to **A**, **B**, and **C**).

Amygdalar sections from seven additional rats with injections of FG into the BF (*n* = 4; R12, R13, R35, R36) or overlying striatum (*n* = 3; R19, R20, R26) were processed for double labeling of FG with markers for one of the three main interneuronal subpopulations of the basolateral amygdala (PV, CR, and SOM) to determine if PV+ and CR+ amygdalar neurons also project to the BF, and whether any of these three neuronal subpopulations project to the striatum (see right sides of Figures 1A and B for the location of these injection sites). No PV+ amygdalar neurons were retrogradely labeled (Figures 10A,B). In nuclei such as the anterior basolateral, where CR is found only in non-pyramidal neurons, no CR+ neurons were retrogradely labeled (Figure 10C). However, in other nuclei such as the BMp, robust CR-ir is found in small non-pyramidal neurons and light CR-ir is found in pyramidal cells, which are considerably larger than the CR+ non-pyramidal neurons (McDonald, 1994; McDonald and Mascagni, 2001). In the latter nuclei, none of the CR+ non-pyramidal neurons, but many of the CR+ pyramidal cells, were retrogradely labeled with injections of FG into the BF (Figure 10D). The distribution of SOM+ LRNP neurons in these cases matched that described above for the triple-labeling preparations. Injections of FG confined to the striatum (R20, R26, R19) did not retrogradely label any non-pyramidal amygdalar neurons expressing SOM, PV, or CR. Thus, the partial involvement of the adjacent fundus striati in some of the injection sites centered in the BF could not have contributed to the FG labeling of LRNP neurons in these cases.

## Discussion

This is the first study to demonstrate that a subpopulation of neurons in the cortical and basolateral amygdalar nuclei that project axons to the BF are SOM+ non-pyramidal neurons, and that there are similar neurons in the ventral portion of the external capsule adjacent to the amygdala and in anterior portions of the medial amygdalar nuclear complex. These “long-range non-pyramidal neurons” (LRNP neurons) constituted a small subpopulation of the SOM+ neurons in each amygdalar nucleus that contained these cells, and they were far outnumbered by SOM-negative retrogradely labeled neurons in these nuclei. Most of the latter neurons in the cortical and basolateral nuclei had pyramidal or piriform cell bodies and appeared to be amygdalar pyramidal cells, the principal neurons of these nuclei (McDonald, 1992a). About one-third of the SOM+ LRNP neurons were CB+ or NPY+, and one-half were GAD+. Non-pyramidal amygdalar neurons containing PV or CR were never retrogradely labeled.

### Comparison with previous studies

The distribution of retrogradely labeled FG+ neurons seen in the present study was very similar to that obtained in a previous study in the rat that utilized wheat germ agglutinin-conjugated horseradish peroxidase (WGA-HRP) as a retrograde tracer (Grove, 1988). Thus, with WGA-HRP injections into ventral portions of the SI, Grove found a diffuse array of retrogradely labeled neurons extending through ventral portions of the amygdala, including the medial, cortical, amygdalohippocampal, basomedial, and basolateral amygdalar nuclei, and in the adjacent ventral portion of the external capsule (see Figure 6 of Grove, 1988). Like our cases R1, R11, and R28, WGA-HRP injections into the dorsal SI also produced retrograde labeling in the central nucleus, particularly its medial subdivision (see Figure 4 of Grove, 1988). Similar to our results, few retrogradely labeled neurons were seen in the lateral nucleus with any SI injections (Grove, 1988). Comparable widespread retrograde labeling was also reported in the monkey with WGA-HRP injections into the nucleus basalis of Meynert (Russchen et al., 1985).

Since the SI/VP region of the rat BF is traversed by amygdalar axons projecting to more rostral forebrain regions such as the olfactory tubercle and prefrontal cortices, it is possible that some of the retrogradely labeled amygdalar neurons seen in our study, and in previous studies with BF injections of retrograde tracers (Russchen et al., 1985; Grove, 1988), could be labeled by uptake of tracer by fibers-of-passage (Lanciego and Wouterlood, 2011). However, the iontophoretic injection of FG through narrow-diameter micropipettes in the present study should minimize uptake by fibers of passage (Schmued and Fallon, 1986; Pieribone and Aston-Jones, 1988; Divac and Mogensen, 1990), and the results of previous anterograde tract tracing studies are consistent with our retrograde findings. Thus, in the autoradiographic studies of Krettek and Price (1978) in the rat and cat, injections of tritiated amino acids into the basolateral, basomedial, medial, and cortical nuclei produced diffuse silver grains, indicative of terminal labeling, that filled the SI/VP region but remained confined to its boundaries. Studies using Phaseolus vulgaris leucoagglutinin (PHA-L) as an anterograde tracer demonstrated significant numbers of labeled axons and terminals in the rat SI/VP region after iontophoretic injections of PHA-L into the basolateral nucleus (Jolkkonen et al., 2002), basomedial nucleus (Petrovich et al., 1996; Jolkkonen et al., 2002), cortical nucleus (Petrovich et al., 1996), medial nucleus (Canteras et al., 1995), and central nucleus (Petrovich and Swanson, 1997; Gastard et al., 2002; Jolkkonen et al., 2002; Loopuijt and Zahm, 2006). Although to the authors' knowledge there have been no previous reports of projections of the rostral intercalated nuclei to the BF in the rat, these projections have been demonstrated in the cat using both anterograde and retrograde tract tracing techniques (Paré and Smith, 1994).

### Long-range non-pyramidal neurons (LRNP neurons) in the corticobasolateral amygdala

Virtually all of the non-pyramidal neurons in the amygdala that had long-range projections to the BF (LRNP neurons) expressed SOM. The great majority of these SOM+ LRNP neurons were seen in the cortical nuclei, basolateral nuclei (consisting of the basolateral, lateral, and basomedial nuclei), and the amygdalohippocampal area. This region of the amygdala, which has been termed the corticobasolateral nuclear complex (CBL), contains neurons that resemble those of the cerebral cortex (McDonald, 1992a, 2003; Sah et al., 2003). The cell types in all of these amygdalar nuclei are similar, but they have been studied primarily in the basolateral amygdala. The principal neurons in the CBL are pyramidal-like projection neurons with spiny dendrites that utilize glutamate as an excitatory neurotransmitter, whereas most non-pyramidal neurons in the CBL are spine-sparse interneurons that utilize GABA as an inhibitory neurotransmitter (McDonald, 1982, 1985, 1992a,b, 1996, 2003; Millhouse and DeOlmos, 1983; Fuller et al., 1987; Carlsen and Heimer, 1988; McDonald and Augustine, 1993). Dual-labeling immunohistochemical studies in the basolateral amygdala suggest that the CBL contains at least four distinct subpopulations of GABAergic non-pyramidal neurons that can be distinguished on the basis of their content of calcium-binding proteins and peptides. These subpopulations are: (1) PV+/CB+ neurons, (2) SOM+/CB+ neurons, (3) large multipolar cholecystokinin+ neurons that are often CB+, and (4) small bipolar and bitufted interneurons that exhibit extensive colocalization of calretinin, cholecystokinin, and vasoactive intestinal peptide (Kemppainen and Pitkänen, 2000; McDonald and Betette, 2001; McDonald and Mascagni, 2001, 2002; Mascagni and McDonald, 2003; Mascagni et al., 2009). In addition, it has been demonstrated that the expression of NPY defines a distinct subpopulation of SOM+ neurons in the CBL (McDonald, 1989; McDonald et al., 1995).

A previous retrograde tract tracing study, with very large injections of HRP and WGA-HRP into multiple rostral cortical and striatal targets of the rat anterior basolateral nucleus (including the medial, lateral, and orbital prefrontal cortex, and the rostral ventral striatum), demonstrated that 85% of the neurons in the BLa were projection neurons whose morphology resembled that of pyramidal cells (McDonald, 1992b). Only 3 out of 1150 HRP-labeled BLa neurons (0.26%) in the four rats examined in that study were small non-pyramidal neurons, and these had very low levels of reaction product. It was unclear whether these lightly labeled neurons were retrogradely labeled, or labeled due to transneuronal transport of HRP (McDonald, 1992b).

Although these findings suggested that most, if not all, of the non-pyramidal neurons in the basolateral nucleus were interneurons, other studies combining retrograde tract tracing with immunohistochemistry for non-pyramidal cell markers indicated that there was a very sparse subpopulation of non-pyramidal cells in the CBL that had projections to extra-amygdalar regions. Thus, a small number of non-pyramidal NPY+ neurons in the lateral nucleus were retrogradely labeled by injections of Fast Blue into the entorhinal cortex (Köhler et al., 1986), a few SOM+ neurons in the lateral nucleus were labeled by injections of WGA-HRP into multiple cortical targets of the CBL (McDonald, 1986), and a small number of SOM+ neurons in the basomedial nucleus were labeled by injections of WGA-HRP into the medial preoptic-hypothalamic region (McDonald, 1987). Collectively, the results of these studies, in conjunction with the present investigation, clearly indicate that a small subpopulation of non-pyramidal cells distributed throughout virtually all portions of the CBL consists of projection neurons rather than interneurons.

Previous studies have shown that NPY is expressed in SOM+ neurons, but not in other non-pyramidal subpopulations of the rat CBL (McDonald, 1989). In fact, virtually all NPY+ neurons in the CBL are also SOM+, which is consistent with the finding of the present study that there were very few LRNP neurons in the CBL that were NPY+ and SOM-negative. Maps of single and double-labeled neurons in SOM/NPY dual-labeled preparations suggest that the majority of SOM+ neurons in the rat basomedial nuclei, cortical nuclei, and ventral external capsule, the main areas containing LRNP neurons, are also NPY+ (McDonald, 1989). The finding of the present investigation that only 35% of SOM+ LRNP neurons projecting to the BF are NPY+ suggests that these LRNP neurons are less likely to express NPY than nonLRNP SOM+ neurons.

CB is expressed in three separate non-pyramidal cell subpopulations in the rat basolateral amygdala: PV+ neurons, SOM+ neurons, and a subset of large CCK+ neurons (McDonald and Mascagni, 2001, 2002; Mascagni and McDonald, 2003). The majority of SOM+ neurons in the basolateral nucleus (91%) and lateral nucleus (67%) express CB (McDonald and Mascagni, 2002). If the majority of SOM+ neurons in the basomedial and cortical nuclei also express CB+, the finding of the present investigation that only 37% of SOM+ LRNP neurons projecting to the BF are CB+ suggests that these LRNP neurons are less likely to express CB than nonLRNP SOM+ neurons. Unfortunately, there have been no studies examining colocalization of NPY with CB in the amygdala, so it is unclear how much overlap there is in between the FG+/SOM+/CB+ and FG+/SOM+/NPY+ LRNP neuronal subpopulations.

The phenotypes of amygdalar LRNP neurons projecting to the SI/VP region of the BF are remarkably similar to those of hippocampal LRNP neurons projecting to the medial septal region of the BF, particularly the LRNP neurons in CA1 of Ammon's horn. Thus, as in the amygdala, more than 90% of the hippocamposeptal LRNP neurons in Ammon's horn are SOM+ (Zappone and Sloviter, 2001; Jinno and Kosaka, 2002; Gulyás et al., 2003; Jinno et al., 2007). Similar to amygdalar-BF LRNP neurons, many hippocamposeptal LRNP neurons in CA1 also express NPY (20–46%) or CB (30–80%) (Tóth and Freund, 1992; Jinno and Kosaka, 2002; Gulyás et al., 2003; Jinno et al., 2007). One big difference in the hippocampus versus the amygdala is that the majority of hippocamposeptal neurons are LRNP neurons (Alonso and Köhler, 1982; Tóth and Freund, 1992; Zappone and Sloviter, 2001), whereas in the amygdala these cells constituted about 5–13% of amygdalar-BF neurons.

In the basomedial and cortical amygdalar nuclei approximately one-half of SOM+ LRNP neurons projecting to the BF were GAD+. Although these results might indicate that there are two distinct subpopulations of amygdalar LRNP neurons, other evidence suggests that the GAD-negative LRNP neurons may merely have levels of GAD that are below the threshold of immunohistochemical detection. Thus, although the cell bodies of hippocamposeptal LRNP neurons in the rat were judged to be GABA-negative, it was noticed that they all appeared to exhibit very faint staining in immunoperoxidase preparations (Tóth and Freund, 1992). Subsequent anterograde tract tracing studies revealed that the axon terminals of these hippocamposeptal neurons were indeed GABA-positive (Tóth et al., 1993). These observations are consistent were numerous studies which have shown that GABAergic neurons with distant projections typically have very low levels of GABA/GAD in their somata due to rapid transport of these neurochemicals to the axon terminals (for a discussion of this issue see Tóth and Freund, 1992). In the mouse hippocampus, which may have higher levels of GABA/GAD than rat (Jinno et al., 1998), prolonged incubation of sections in GAD antibody (10 days) demonstrated that all hippocamposeptal LRNP neurons were indeed GAD+. In the present study, colchicine-injection and a longer than usual incubation in GAD antibody (four days) was used to increase GAD staining in neuronal somata. Nevertheless, the failure to reliably stain the somata of GABAergic projection neurons in the central and medial amygdalar nuclei (McDonald and Augustine, 1993; Poulin et al., 2008) clearly indicates that these technical measures fell short of their goal to identify all GABAergic amygdalar neurons. It therefore seems possible that the great majority of amygdalar LRNP neurons projecting to the BF are GABAergic, like those of the hippocampus, but that many have very low levels of GAD. Future retrograde tract tracing studies, perhaps using GAD-green fluorescent protein knock-in mice as experimental animals to better visualize all GABAergic neurons (Tamamaki et al., 2003), will be required to resolve this issue.

### Long-range non-pyramidal neurons in the medial nucleus and external capsule

A few SOM+ neurons in the anterior portion of the medial nucleus were retrogradely labeled by BF injections of FG. Since the cell types in the medial nucleus are not cortex-like as in the CBL complex, spiny pyramidal and spine-sparse non-pyramidal neurons cannot be recognized in this nucleus (Hall, 1972; McDonald, 1992a). The high density of SOM+ neurons in the medial nucleus, and the fact that many of these cells take part in its projections to the medial preoptic-hypothalamic region, suggests that most of these cells are a subpopulation of the principal neurons in this nucleus (McDonald, 1987, 1989; Równiak et al., 2008). The present investigation indicates that a very small subpopulation of these SOM+ neurons project to the BF.

Many of the LRNP neurons labeled by BF injections of FG were found in or adjacent to the white matter of the ventral portion of the external capsule that separates the amygdala from the piriform cortex. Likewise, most of the hippocamposeptal LRNP neurons in Ammon's horn are found in the stratum oriens, which lies adjacent to the white matter of the alveus (Zappone and Sloviter, 2001; Jinno and Kosaka, 2002; Gulyás et al., 2003; Jinno et al., 2007). Similarly, there is an extensive array of GABAergic LRNP neurons in or adjacent to the white matter of the entire neocortex that project to both neighboring as well as distant areas of the cortex (Tomioka et al., 2005; Tomioka and Rockland, 2007; Clancy et al., 2009). Like the LRNP neurons of the amygdala and hippocampus that project to the BF, virtually all of the neocortical LRNP neurons are SOM+ and also express NPY (Tomioka et al., 2005; Tomioka and Rockland, 2007). Thus, the SOM+ LRNP neurons seen in the region of the ventral portion of the external capsule in the present study appear to be part of this white matter-associated system of neurons, but they also have projections to the BF. In addition to their long-range projections, the LRNP neurons associated with the white matter of the neocortex have reciprocal connections with the area of neocortex that overlies them (Meyer et al., 1991; Shering and Lowenstein, 1994; Clancy et al., 2001). This suggests that the SOM+ LRNP neurons seen in the region of the ventral portion of the external capsule in the present study may have reciprocal connections with the adjacent piriform cortex or the cortex-like CBL nuclei of the amygdala, in addition to their connections with the BF.

### Functional significance

The results of this investigation, along with previous tract tracing studies, demonstrate that there are multiple excitatory and/or inhibitory projections to the BF from the amygdala. The excitatory amygdalar inputs to the BF appear to arise from pyramidal neurons in the CBL nuclear complex. The projections from the basolateral portion of this complex have been shown to innervate cholinergic BF neurons via asymmetrical (presumably excitatory) synapses (Záborszky et al., 1984). Previous studies demonstrated that inhibitory inputs arise from the central nucleus and intercalated nuclei. Thus, the central nucleus has projections to the SI that mainly target noncholinergic neurons, but also synapse with a distinct subpopulation of small cholinergic neurons, primarily via symmetrical (presumably inhibitory) synapses (Gastard et al., 2002; Jolkkonen et al., 2002; Loopuijt and Zahm, 2006). The rostral intercalated nuclei have strong GABAergic projections to the SI that form symmetrical synapses with nonGABAergic, presumptive cholinergic neurons (Paré and Smith, 1994). Since at least half of the LRNP neurons in the CBL complex and external capsule are GABAergic, the present investigation demonstrates that they comprise another inhibitory projection to the BF. Although only a small number of these neurons were seen in each section, they collectively constitute a major inhibitory input to the BF.

An understanding of the role of the amygdalar-BF LRNP neurons will require knowledge of their inputs, as well as their targets in the BF. Hippocamposeptal LRNP neurons mainly target GABAergic neurons in the BF that have projections back to the hippocampus (Tóth et al., 1993). The latter neurons target many types of hippocampal GABAergic neurons including the hippocamposeptal LRNP neurons (Takács et al., 2008). This reciprocal inhibitory loop in the hippocamposeptal network appears to be critical for the generation of rhythmic oscillatory activity involved in mnemonic function (Buzsáki and Chrobak, 1995; Freund and Buzsáki, 1996; Dragoi et al., 1999; Buzsáki, 2002; Wang, 2002; Jinno et al., 2007). GABAergic neurons in the SI/VP portions of the BF have projections to the amygdala (Mascagni and McDonald, 2009), but they mainly innervate PV+ interneurons (and also pyramidal cells) in the basolateral nucleus (McDonald et al., 2011) rather than the SOM+ LRNP neurons that predominate in the basomedial nucleus, cortical nucleus, and external capsule. Thus, the point-to-point reciprocal inhibitory loop important for hippocamposeptal network functions appears to be lacking in the amygdala. As a result, although oscillatory activity in the amygdala is important for emotional arousal and emotional memory (Paré and Collins, 2000; Paré et al., 2002; Pape et al., 2005; Lesting et al., 2011), it is not clear at this point how the connections of the amygdalar LRNP neurons projecting to the BF would permit them to contribute to oscillatory activity in a manner similar to that seen in the hippocampus. Stimulation studies of the amygdala have shown that neocortical desynchronization can be obtained with stimulation of the lateral, basolateral, and central nuclei, but not with stimulation of the basomedial or cortical nuclei (Kaada, 1972). It remains to be determined if this differential effect is related to the presence of inhibitory LRNP neurons in the latter nuclei.

### Conflict of interest statement

The authors declare that the research was conducted in the absence of any commercial or financial relationships that could be construed as a potential conflict of interest.

## Abbreviations

ac: anterior commissure
ACo: anterior cortical nucleus
AHA: amygdalohippocampal area
BAOT: bed nucleus of the accessory olfactory tract
BF: basal forebrain
BLa: anterior subdivision of the basolateral nucleus
BLp: posterior subdivision of the basolateral nucleus
BMa: anterior subdivision of the basomedial nucleus
BMp: posterior subdivision of the basomedial nucleus
CB: calbindin
CBL: corticobasolateral nuclear complex of the amygdala
CL: lateral subdivision of the central nucleus
CLC: capsular subdivision of the central nucleus
CM: medial subdivision of the central nucleus
CP: caudate-putamen
CR: calretinin
cst: commissural stria terminalis
ec: external capsule
FG: Fluorogold
fx: fornix
GAD: glutamic acid decarboxylase
GP: globus pallidus
HDB: horizontal limb of the nucleus of the diagonal band
IN: intercalated nuclei
L: lateral nucleus
Ld: dorsal subdivision of the lateral nucleus
LRNP neurons: long-range non-pyramidal neurons
Lv: ventral subdivision of the lateral nucleus
Mad: anterodorsal subdivision of the medial nucleus
Mav: anteroventral subdivision of the medial nucleus
MCPO: magnocellular preoptic nucleus
Mpd: posterodorsal subdivision of the medial nucleus
Mpv: posteroventral subdivision of the medial nucleus
NPY: neuropeptide Y
PV: parvalbumin
PLCo: posterolateral subdivision of the cortical nucleus
PMCo: posteromedial subdivision of the cortical nucleus
PC: piriform cortex
SI: substantia innominata
SOM: somatostatin
VP: ventral pallidum.
